# Managing Critical Patient-Reported Outcome Measures in Oncology Settings: System Development and Retrospective Study

**DOI:** 10.2196/38483

**Published:** 2022-11-03

**Authors:** Olga Strachna, Onur Asan, Peter D Stetson

**Affiliations:** 1 School of Systems and Enterprises Stevens Institute of Technology Hoboken, NJ United States; 2 Division of Digital Products and Informatics Memorial Sloan Kettering Cancer Center New York, NY United States

**Keywords:** patient-reported outcome measures, delivery of health care, self-report, quality of care, neoplasms, surveys and questionnaires, clinical decision support systems

## Abstract

**Background:**

Remote monitoring programs based on the collection of patient-reported outcome (PRO) data are being increasingly adopted in oncology practices. Although PROs are a great source of patient data, the management of critical PRO data is not discussed in detail in the literature.

**Objective:**

This first-of-its-kind study aimed to design, describe, and evaluate a closed-loop alerting and communication system focused on managing PRO-related alerts in cancer care.

**Methods:**

We designed and developed a novel solution using an agile software development methodology by incrementally building new capabilities. We evaluated these new features using participatory design and the Fit between Individuals, Task, and Technology framework.

**Results:**

A total of 8 questionnaires were implemented using alerting features, resulting in an alert rate of 7.82% (36,838/470,841) with 13.28% (10,965/82,544) of the patients triggering at least one alert. Alerts were reviewed by 501 staff members spanning across 191 care teams. All the alerts were reviewed with a median response time of 1 hour (SD 185 hours) during standard business hours. The most severe (red) alerts were documented 56.83% (2592/4561) of the time, whereas unlabeled alerts were documented 27.68% (1298/4689) of the time, signaling clinician concordance with the alert thresholds.

**Conclusions:**

A PRO-based alert and communication system has some initial benefits in reviewing clinically meaningful PRO data in a reasonable amount of time. We have discussed key system design considerations, workflow integration, and the mitigation of potential impact on the burden of care teams. The introduction of a PRO-based alert and communication system provides a reliable mechanism for care teams to review and respond to patient symptoms quickly. The system was standardized across many different oncology settings, demonstrating system flexibility. Future studies should focus on formally evaluating system usability through qualitative methods.

## Introduction

### Background

Patient-reported outcomes (PROs) are being increasingly collected as a part of routine clinical care, capturing patients’ self-reported symptoms, function, and quality of life. They support the goal of facilitating clinician-patient communication, mutual understanding of patient preferences, and enabling shared decision-making with an impact on treatment decisions [1-7]. PRO data collection is particularly significant for the oncology patient population, especially for patients on clinical trials, because of the critical need to track symptomatic adverse events related to cancer treatments, which have a significant impact on the clinical outcomes and quality of life of patients [5,8-14]. In addition, PROs are relied on for managing health care use [4,8,11]. PRO data are only valuable as long as patients complete the surveys accurately and timely. Limited survey completion rates minimize the ability to draw clinical conclusions from sparsely filled out data [15]. Studies have shown that clinician engagement in the process of administering PRO programs via patient education or outreach has a direct positive impact on patient engagement [16-18]. Provider disengagement in the process of reviewing the data may disincentivize patients from completing their PRO assessments [19]. Therefore, it is essential for clinicians to follow-up with patients regarding any significant outcomes reported in a streamlined and timely manner.

As with many other clinical applications, there has been an interest to integrate PROs within clinical workflows; however, evidence shows limited success [14,20-24]. Sources of patient-generated health data such as PROs are relatively new to the standard of care practices, and there is not always a standard mechanism in place for clinicians to handle PROs appropriately with varying implementation strategies [9]. At the same time, notifying clinicians about all patient responses does not always result in a timely follow-up with the patient if there are workflow barriers impeding communication, such as reviewing too many PRO responses [25-27].

The concept of alerting is not new to health care, with long-standing applications in clinical decision support systems for drug-drug interactions [28,29], adverse event monitoring [20,30,31], abnormal laboratory results [25,32-36], and many others [37]. The idea of PRO alerts is distinct from the standard clinical alerts mentioned, in that it involves asynchronous interruptive and noninterruptive communication between patients and care team members as well as coordination among care team members within the system. Several studies have mentioned using alert-based features within the context of PROs; however, none of them have discussed the communication aspect with patients, analyzed the impact on workload, and described the detailed designs of such alerting systems [8,9,38-40].

Given the rise in the popularity of remote monitoring programs, including the use of PRO data during the COVID-19 pandemic, there have been several enthusiastic studies on program evaluations, and remote monitoring programs are expected to increase in adoption in the post–COVID-19 pandemic years [41-44]. Remote monitoring programs are novel in and of themselves; therefore, as a part of this study, it was important to consider the design aspect of a work management system to handle critical results in a timely manner. In addition, it was critical to understand the impact of running such programs asynchronously from clinical visits to allocate appropriate resources to respond to patient-specific needs outside standard staffed business hours, with implications for program monitoring and management.

### Objectives

To date, there has been pervasive interest in using PROs for remote symptom management in oncology standard of care practice, but very little is known about the management of critical patient symptom responses and the engagement of clinical staff in the review of patient responses to address them appropriately. Given the increasing adoption of PROs in standard oncological practices, we identified a need to design a robust PRO alert management and communication system that scales with increasing clinic patient volumes and patient demand for asynchronous communication. Considering the potential clinic disruption, it was important to quantify the impact of such a new system on clinic workloads. For this study, we designed and implemented the alert and communication system separate from the electronic health record (EHR) but with a tight integration of key results. It was unknown what features would be needed in such a system and whether staff adoption of and engagement with such a system would be successful. The findings presented in this paper provide insights into the architectural design and a detailed list of features for any organization considering implementing a mechanism for handling the critical PROs reported.

In this paper, we present the results of our PRO-based alerting and communication system design, summarize key quantitative results, and reflect on the implications of scaling the adoption of this technology more widely. To our knowledge, this is the first paper to report on the design, implementation, and use of a closed-loop alert management and communication system specifically for managing PRO data in cancer care.

## Methods

### Ethics Approval

This retrospective cohort study was approved by the Memorial Sloan Kettering (MSK) Cancer Center institutional review board (approval number 19-090) to be conducted between September 2016 and January 2021.

### Setting

The study was conducted at a high-volume National Comprehensive Cancer Network in and near the New York City area, across all sites of care, including ambulatory care clinics, inpatient services, ambulatory surgery centers, inpatient surgery, and urgent care. The PRO data collection and alerting system was implemented as a standard of care for multiple cohorts of patients with cancer through individually managed PRO programs consisting of interdisciplinary clinical, administrative, and technical teams. Notably, the novel COVID-19 screening questionnaire and COVID-19 symptom questionnaires were administered to virtually all patients coming to MSK for any appointment. Patients enrolled in these programs would have characteristics similar to those of patients who were more prone to receiving cancer treatments. All numerical results reported were for the entire study period, between September 2016 and January 2021. The median age of the patients was 61 years, and overall instrument compliance was 36.89% (447,562/1,213,271) across all patient cohorts that were part of this study.

### Engage System Overview

**Figure 1:**
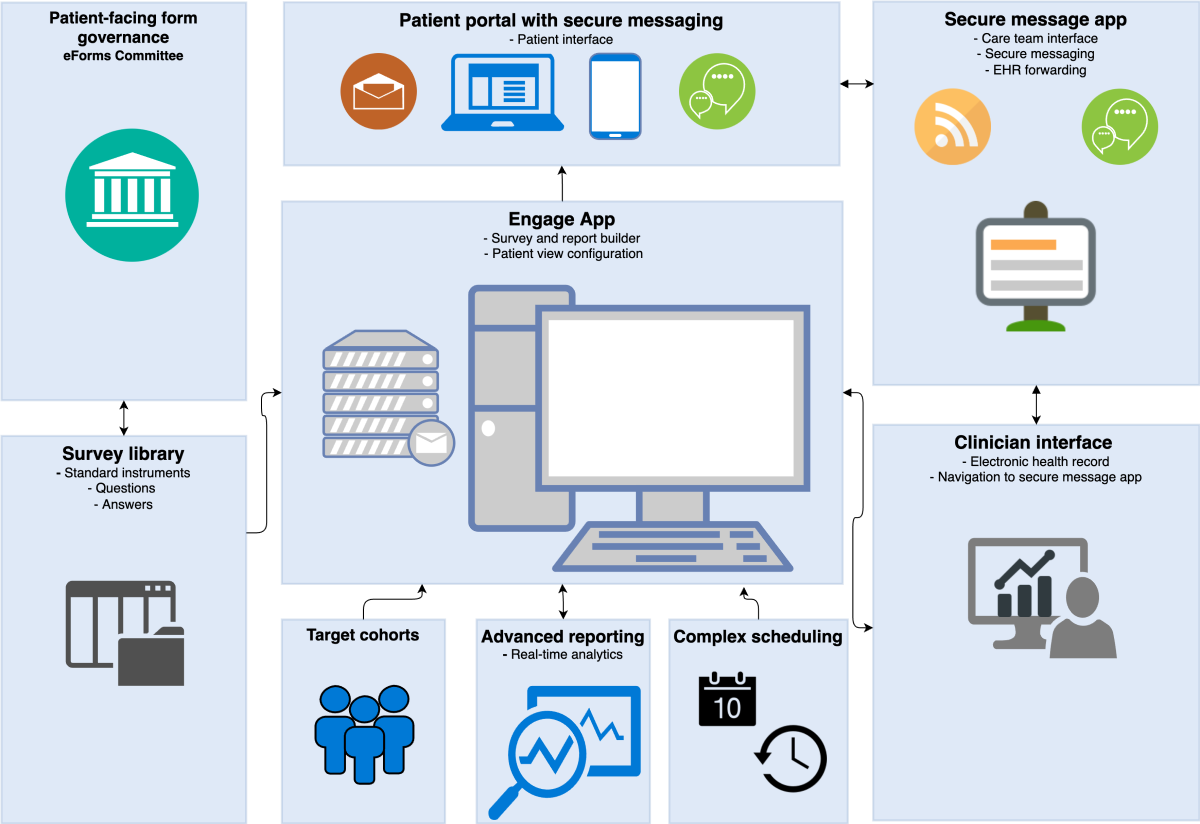
Overview of the homegrown patient-reported outcome (PRO) system called Engage. EHR: electronic health record.

From September 2016 to August 2017, we launched a pilot where we added alerting features incrementally into Engage, our PRO app, and by rolling out the Recovery Tracker, an electronic postoperative symptom survey based on the PRO–Common Terminology Criteria for Adverse Events [45] that is assigned for 10 days after ambulatory surgery cases. The surveys were completed by patients at home via the patient portal account either through a web browser or our mobile app. Engage, although not the focus of this study, was the foundational backbone for the alert notification and communication system discussed in this study. We launched a total of 86 PRO survey instruments in Engage, including standard of care forms such as intake forms, screening questionnaires, short-term symptom assessments, long-term follow-up questionnaires, and research-based questionnaires. Engage was developed as a stand-alone app; it is tightly integrated within MyMSK (developed internally at MSK patient portal), patient-clinician secure messaging system, and the EHR system [7,46]. Engage is depicted in Figure 1 and consists of 4 key subsystems (survey configuration and deployment, alert notification system, patient and clinician user interfaces, and secure messaging system). Engage is further integrated with upstream databases to support cohort identification and scheduling. It is also integrated with downstream clinical information and documentation systems within the existing clinician and support staff workflows to support clinical charting and escalation workflows. In addition to the technical aspects, the system consists of a governance committee overseeing key design and program decisions called the eForms Committee. The focus of this study is to demonstrate the process behind the design, development, and implementation of the alert notification system and its integrations with upstream (ie, survey library, target cohorts, and complex scheduling) and downstream systems (ie, EHR). The design of Engage (our PRO system) is beyond the scope of this study.

### System Governance

To drive decisions and formal governance of the alert notification system, we leveraged our electronic forms committee (eForms Committee), which met monthly. The committee was established at the launch of our PRO initiatives in 2016 to oversee PRO instrument development, evaluate patient burden, discuss impacts on clinician workflow, review regulatory and legal implications, and approve significant changes to the features that were requested by clinical user groups. It is a multidisciplinary committee consisting of health informatics specialists, app development team members, patient engagement specialists, clinicians, researchers, biostatisticians, health information management staff, and hospital administration. In addition to the eForms Committee, PRO work groups were created for each survey instrument, which met more frequently to discuss the management and implementation considerations of the Engage system and provide frequent feedback on system design proposals. PRO work groups also met to decide on setting the initial alerting criteria and adjusting thresholds as needed.

### Steps for Alert Notification System Design

We sought to design and implement a robust and agile PRO-based critical results alert system that notifies the patient and the entire care team of a clinically meaningful patient response as it happens in real time at an oncology care setting. One of the goals for the design was to provide the ability to facilitate secure nonstructured communication between the patient and care team about the abnormal results and to resolve it within the same workflow. A secondary objective of this study was to describe alert volumes, response times, and triaging patterns to understand the implications for scale and feasibility of implementing PROs system wide for all patients with cancer. In addition to the system design and implementation, we created a feedback and governance structure around system enhancements and content revisions based on the Scaled Agile Framework. The feedback was gathered through a series of regular meetings with all project stakeholders including end users, program managers, clinicians, and system developers.

Owing to the lack of an existing methodology for the management of PRO related to critical values (eg, a very severe symptom being reported several days after a surgical event), we referenced models of critical result communication based on abnormal laboratory or radiological findings by reviewing the literature to determine an initial set of desirable system components and features for our PRO-based alert notification system. We conducted a literature review of the existing clinical decision support interventions in PubMed to identify key system components that were necessary to enable clinical alert generation and management. The sample search terms included “critical alert management,” “clinical alert notification,” “critical result notification,” and “abnormal result management.” We identified 5 key capabilities, which were enabled in our alert notification system: alert rule configuration, alert messaging, acknowledgment, triage, and alert export for documentation in the EHR. Once the alerting components were enabled during the pilot, they were adopted by 7 other PRO-based projects, as described in more detail in Multimedia Appendix 1.

Overall, 8 questionnaires were configured with alerting functionalities, targeting more acute symptom assessments, following a recent clinical event that served as a trigger in the target cohorts. In these scenarios, MSK’s best practice expectation was a call back within 2 business days after the clinical event, which would be supplemented with automated symptom assessments. The patients in these cohorts were defined as those who have recently had a surgical event, radiation treatment, chemotherapy treatment, or COVID-19 diagnosis.

**Figure 2:**
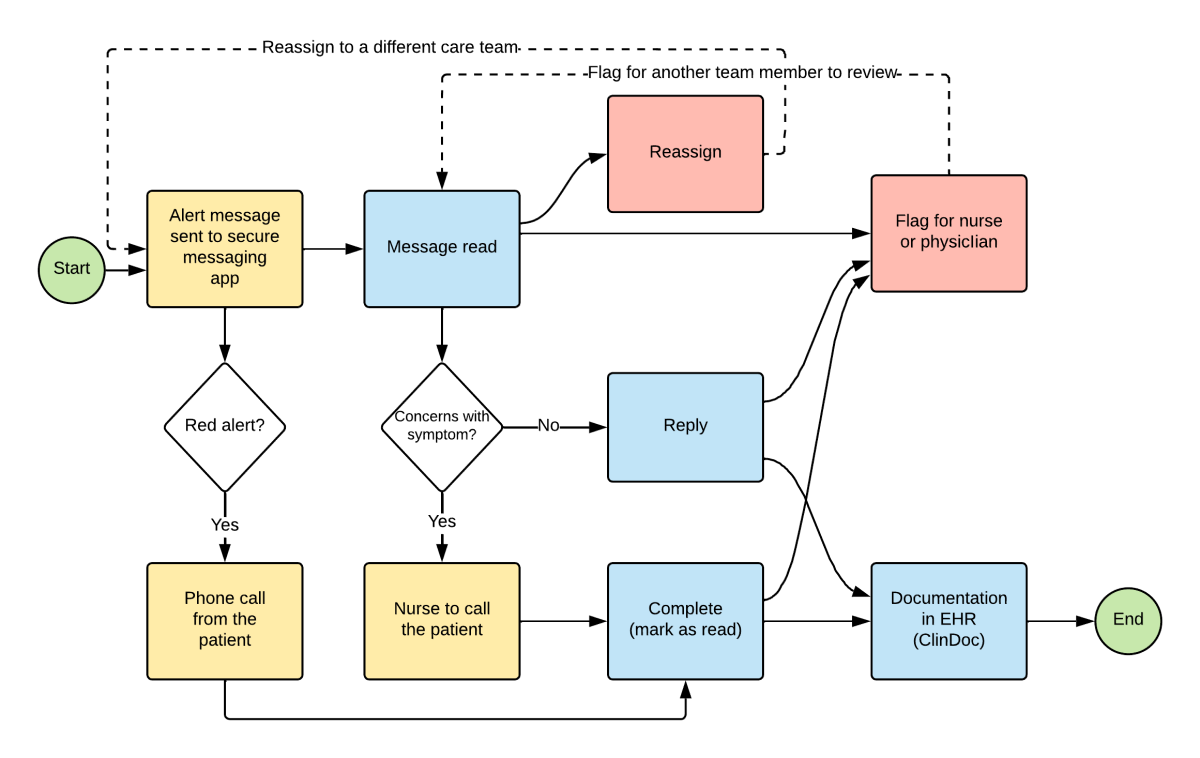
End-to-end processes for alert message management. EHR: electronic health record.

Figure 2 shows the alert management workflow followed by the care team members. An alert was defined as a notification that went out to the care team members because of a survey submission by the patient. A patient could report multiple alerting events in surveys that were designed to be recurring for several days (eg, 10 events in a row for a 10-day survey; each survey can result in an alert). In addition, we implemented a patient-facing alert notification, whereby a patient was notified when their responses triggered a concerning symptom via a pop-up on their screen. The notification advised the patient to call their physician’s office if they were concerned about the symptom worsening. At the same time, this triggered an alert message to be sent to their physician’s inbox. Upon reviewing the message, a care team member had the option to call the patient directly to follow-up on any concerning symptoms or reply to the message. Then, they also have the option to flag the message as an escalation indicator for a more senior care team member. Standard nurse phone calls with patients undergoing oncology treatments included questions about any follow-ups after the treatment (eg, symptom assessments and clarifications about PRO responses that may be concerning or need to be elaborated on). Nursing teams also handled triage of any patient concerns as they arose during phone call conversations, including providing patient education materials, facilitating referrals for prescription refills, and referring patients to urgent care facilities or specialty treatment referrals. The decision to call the patient was based on the guidance established by each clinic and the clinical judgment of the care team members. Finally, users have the option to send the message to the EHR to further document it in a clinical note by clicking the “ClinDoc” button.

### Applying the Fit Between Individuals, Task, and Technology Framework

During the pilot period between September 2016 and August 2017, we participated in biweekly PRO work group meetings consisting of frontline staff, including physicians, nurses, and advanced practitioners; office staff; and administrative staff to solicit ideas about the system features desired by the care team members reviewing the critical PRO results and communicating with patients. We mapped these features in the Fit between Individuals, Task, and Technology (FITT) framework. We selected the FITT framework because it considers the sociotechnical aspects of a successful system adoption, enabling us to understand the attributes of users, technology, and tasks leading to successful adoption. It also allowed us to consider the interaction of all 3 attribute types to envision a more holistic solution. We continued to use the framework throughout the implementation period to elucidate additional features that were important to consider about the tool, task, and person performing the task for each component of the critical result notification framework identified specifically for PROs [47]. In addition, we discussed workflow aspects of the management of symptom alerts and clinical decision-making processes. Through these meetings, we elucidated the key person and task attributes of designing a PRO-focused alert management system. We presented the model to our biweekly informatics working group consisting of informaticians, system developers, and product managers, where we discussed the task- and tool-related attributes of these capabilities. After each feedback session, we documented features, success criteria, and interventions against the key system capabilities identified in the previous section into a FITT framework, charting them into tool-, task-, and person-related buckets.

### Data Collection and Analysis

System use data were collected by querying the underlying reporting database collecting the following variables (defined in Textbox 1) for data between September 2016 and January 2021: patient adoption, patient engagement, alert volume, alert rate, alert types, messaging status, triaging and escalation flags, response times, and clinician involvement. Data were queried using DBeaver software (DBeaver Corporation). Descriptive statistics and data visualizations were developed using Tableau software (Tableau Software, Inc).

**Description of the variables used for analysis.**

**Variable name and definition**

- Patient adoption
  The total number of patients that completed a patient-reported outcome questionnaire and volume of questionnaires completed
- Patient engagement
  The percentage of patients who completed a questionnaire out of the total numbers of questionnaires that were assigned per patient
- Alert volume and rate
  The total number and rate of alerts that were fired per alert-eligible questionnaire
- Alert type
  The severity level of the alert message (red [severe], red-yellow [severe and moderate], and yellow [moderate]) including unlabeled messages (shown as alert)
- Message status
  The final status of the alert message in the secure messaging system (read, replied, completed, and documented).
- Message reassignment flag
  An indicator of whether a message has been reassigned to someone else
- Message escalation flag
  An indicator of whether a message has been flagged for review by another care team member (either a registered nurse or physician)
- Response time
  The time between when the alert message was created to when it was last updated by a care team member (in hours)
- Clinician involvement
  The total number of care teams that were assigned alert messages as measured by unique care team inboxes, including the total number of individual care team members who reviewed, responded to, or handled the alert messages within the care team inboxes

## Results

### Overview

The findings explain how our system was designed, features identified within the FITT framework, how our system creates and schedules alerts, the management and delivery of the alerts, and descriptive statistics of alert management and adoption by the care team members.

### Alert Notification System Components and Features

**Figure 3:**
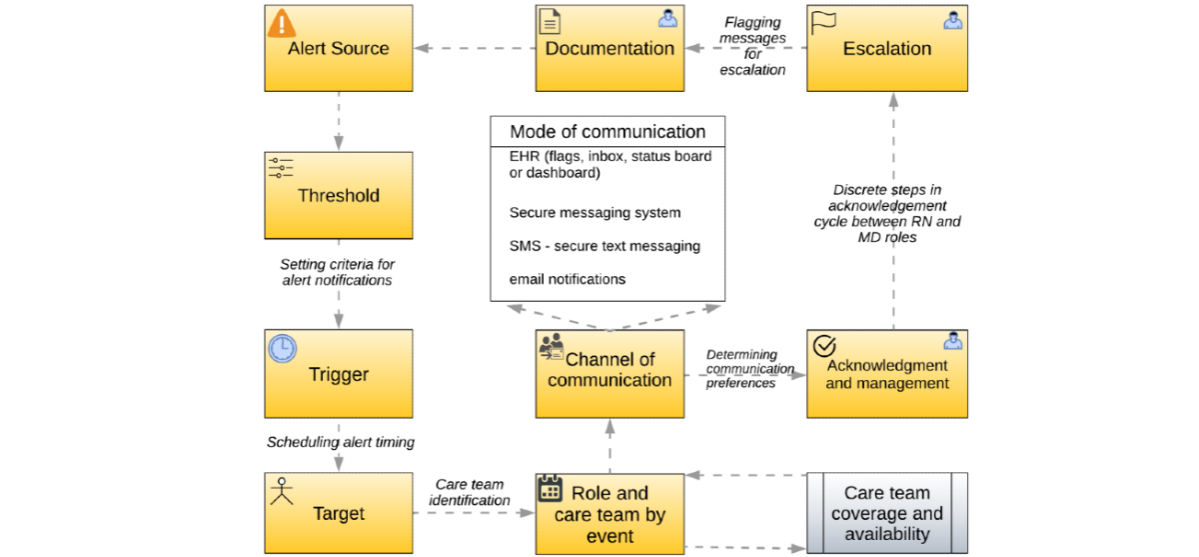
Patient-reported outcome alert system capabilities and process flow. EHR: electronic health record; MD: Medical Doctor; RN: registered nurse.

The main system framework components for identifying and communicating critical PRO responses, which we evaluated against the FITT framework and subsequently implemented in our production PRO tool within our patient portal and secure messaging system, are illustrated in Figure 3 and described in Textbox 2.

Figure 3 depicts the overview of the alerting system at a high level. Starting with the source system, Engage, where questionnaires are built, the alerts are configured and patient survey responses are captured and stored. Once a patient submits a questionnaire, the responses are reviewed by a listener to see whether they pass a predefined threshold. Then, when a trigger event specifying the timing of the alert message is detected, the target recipient is identified (this is captured upfront in the patient cohort definition stage based on coverage), and the message is routed to the appropriate communication channel. In our case, this was routed to a secure messaging system, but we configured for an omnichannel communication strategy. The message is sent to a mailbox and reviewed by the care team members, who have the option to escalate it to other care team members or document the conversation in the downstream EHR clinical note.

**Key components of the alert notification system and their descriptions.**

**Key component and description**

- Alert source and threshold
  Establish a scoring algorithm based on a single response value or a combination of response values to flag a notification to be sent based on a specific patient submission. One submission can consist of multiple question responses (eg, severe pain and severe fatigue). Responses were presented in sections and color coded to show the most critical alerts first in red, followed by responses that were not critical in yellow so that the care team could triage and prioritize their responses to patients.
- Trigger
  Define a technical method to schedule the notification to be sent when a patient reports a certain value. Alert messages were stored as JSON objects, and the notification was done in real time for critical alerts.
- Target
  Define the target system where the message will be visible by the entire patient care team and identify who will receive and manage the message based on their specific clinical role, care relationship with patient, coverage, and availability.
- Communication mode
  Determine the specific alert communication preferences based on a clinician’s role or their tool preference. The communication tools of choice of care team members often varied based on their clinical role. The app accommodated multiple communication modes and the ability to honor the preferred method of communication of each user.
- Acknowledgment and escalation
  Identify discrete steps in the acknowledgment cycle, including escalation of messages to senior clinical roles for more critical follow-ups. Buttons were created to manage each discrete step in the acknowledgment and escalation processes.
- Documentation
  Record the most recent status of an alert in the source system, including escalation, or document the follow-up with the patient in the medical record.

### FITT Framework Results: Alert Features by Person, Task, and Tool

After we attended biweekly PRO working groups, we charted the desired features and user needs into a modified FITT framework that was stratified by each major component identified in the literature review, as summarized in Textbox 2. This resulted in a comprehensive list of program management processes and system requirements and informed our user acceptance testing scenarios during the development and subsequent rollout of the app. The results are summarized in Table 1.

**Table 1:** Summarizing capabilities by the tool, task, and person features of our alert system.

Component	Tool features	Task features	Person features
Alert source authentication	• Care team tracking system in place • Identify systems for generating and receiving notifications • Single sign on to all applications	• Navigation between apps used to respond to an alert • User account verification and management	• Care team identification and authentication to all systems • Patient access to survey submission tool to submit and review patient alerts • Training of users to access and navigate between systems
Alert creation	• Define the customizability points of an alert (frequency, mode, method, and target [person]) • Enable a rules engine to define threshold setting rules and optimization • Bundled alert creation	• Establish trigger points • Identify alert severity levels • Analyze data and tune thresholds for triggers • Define workflows by alert severity (urgent, semiurgent, or nonurgent)	• Governance for creating, reviewing, and tuning alert triggers and thresholds • Patient knowledge of alert creation
Communication	• Interoperable modes of communication established (EHR^a^, patient portal, SMS text messaging, apps, email, and telecom [pager, Vocera, telephone, and e-fax]) • Manage preferences for the mode of alert communication	• Redundancy management • Alert bundling and sorting based on similar alerts • Develop definitions of severity language	• Availability and coverage of the care team • Notification preferences established (tool of preference to log into for alerts) by event type or service or patient procedures • Digital communication between the care team and patient
Reminders and escalation	• Method in place to set reminder schedule for critical alerts if they have not been reviewed • Autoescalation of alerts that have not been reviewed	• Rules for reminders • Rules for escalation • Autoescalation • Due date escalation • Missed alert handling	• Department-specific training on acknowledgment management and follow-up actions • Monitoring of escalation patterns
Acknowledgment and management	• Identify systems receiving acknowledgment • Rerouting of messages • Method in place for handling errors in alert creations and communication • Ability to acknowledge a bundle of alerts • Autoacknowledgment	• Define actions that reflect acknowledgment (time, action, and role) • Prioritization based on severity • Voluntary forwarding of alerts • Handling errors in communication	• Training to the care team members on acknowledgment management and follow-up actions • Monitoring of acknowledgment rates
Documentation	• Ability to document alert summary and resolution findings • Ability to copy and paste alert message contents into a clinical note	• Define the levels of documentation to close loop on alert • Feature to import alerts into EHR templates	• Define documentation reviewers • Documentation workflows defined to the care team members, specific to each service and survey

^a^EHR: electronic health record.

### Alert Creation and Scheduling

**Figure 4:**
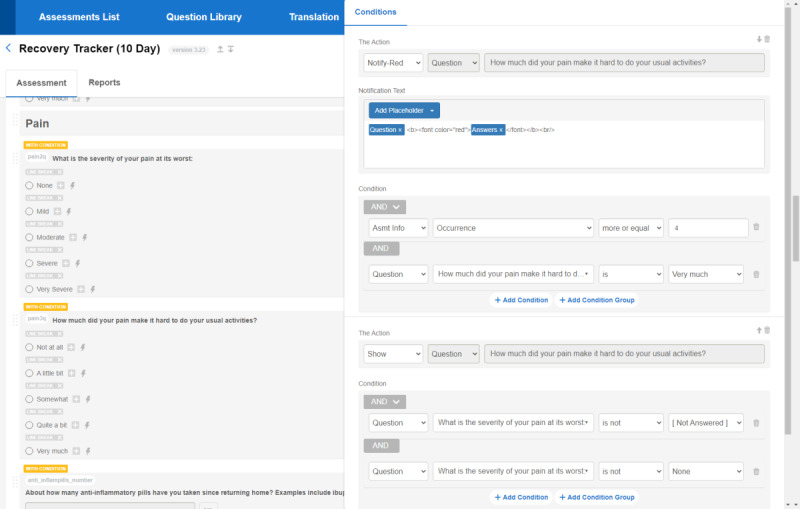
Screenshot of the survey library app, specifically the alert threshold configuration interface.

After alert rules had been established by PRO work groups consisting of the most up-to-date clinical standard of practice guidelines adopted by each service, the system administrators were responsible for implementing the criteria. Alert creation was accomplished with a configuration tool in the alert source system, allowing system managers to configure complex rules based on patient responses to individual questions or a combination of questions. The care teams also requested the ability to specify distinct alert rules for specific clinical contexts (such as triggering an alert based on a specific surgical procedure, diagnosis, or treatment regimen) and to vary based on the time span between the clinical event and the time the questionnaire was completed by the patient (such as not firing an alert for pain reported one day after surgery and fire starting after day 3). The alert configuration component, depicted in Figure 4, is where the system administrators configure the subject of the alert message and body of the notification message, including the ability to specify severity levels using visual color indicators and other HTML and cascading style sheets–based text formatting options of the message body. The color label feature was requested by clinicians after spending a few months responding to nonlabeled messages as a mechanism to emphasize severity. Adding color labels that indicate the level of severity to the subject of the message allowed the care team members to triage these notifications appropriately. There was also an ability to integrate the clinical context into the body of the alert notification. Once the alert rules were configured at the questionnaire level, setting a threshold and directionality (greater, equal to, or less than) was the next capability, defined as the level that must be crossed when an alert fires. Next, we developed a triggering mechanism, which is a technical method to configure and synchronize the schedule of sending the notifications based on business rules.

### Alert Delivery and Management Workflow

**Figure 5:**
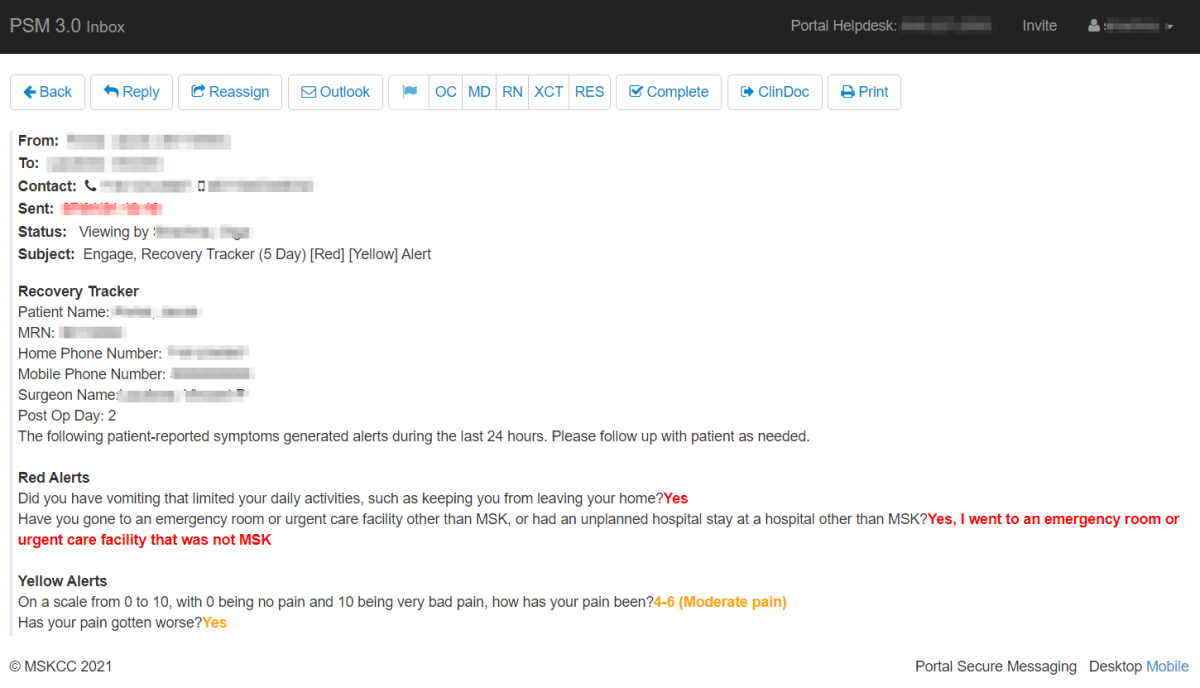
Screenshot of the patient-facing and clinician-facing alert messages. MSK: Memorial Sloan Kettering.

Once the alert message is ready to be delivered to a target (defined as a primary clinician taking care of the patient at the time the survey was assigned), there is an ability for the rest of the care team members to subscribe to the primary clinician’s secure inbox to review and respond to patient messages on behalf of the entire care team. A screenshot of the alert message is shown in Figure 5. This inbox is built within our patient portal, where the staff can securely communicate with patients bidirectionally. The primary users of the inbox were nursing and administrative office staff supporting the clinic. While in the inbox, users can reassign the message to a different provider if someone else is covering this patient. After opening the message, the staff can acknowledge the message by marking it as complete, reply to the patient directly, or escalate the message to the clinician’s office staff, typically a nurse. In addition to the digital workflow, the staff can take the manual route by following up with the patient via a phone call and marking the message as complete. Once an action is taken on the alert message, users have the option to send the message thread to the EHR so that it can be imported into a note. This last import step closes the loop on the alert management life cycle.

Figure 5 demonstrates the output of the alert configuration, which is the message that shows up in the care team’s inbox. The message subject indicates the alert severity levels, and the body contains the red or yellow symptom indicators, showing which symptoms triggered the alert. The message body also includes some contextual information about the patient along with their contact information, if available, so that care team members can reach out directly if the message is urgent. The message controls are available on top, supporting the ability to reassign to a different care team member, reply directly to the patient, forward the message to email, flag the message to a different person by role, mark the message as complete, send the message to the EHR (ClinDoc), and finally print the message.

### Adoption by Numbers

Through the 8 questionnaires configured with the alerting feature, 7.82% (36,838/470,841) alerts fired per completed symptom assessment, and 13.28% (10,965/82,544) of the patients fired at least one alert out of all patients who received at least one survey. This means that 92.28% (434,003/470,841) of the patient surveys did not trigger an alert; therefore, their responses did not need to be reviewed by the care teams. The alerts were managed by 191 different care teams consisting of 501 staff members. Each care team configured its own mailbox, which was set up at the physician clinic, service, or clinic location level. Staff members subscribe to a care team mailbox and have access to review patient messages, triage them, and respond to the patients. A median of 5 staff members managed each mailbox, with care team volumes ranging from a minimum of 1 to a maximum of 75 staff members. The median number of alerts per care team inbox was 35 (95% CI 0-145).

### Alert Management Patterns

Regarding message triage and escalation, 4.4% (1631/36,838) of the alerts were reassigned to other care teams, and 16.7% (6156/36,838) of the alerts were flagged for another care team member to review. The care team members who received the reassigned messages were often located at different campuses, closer to the patients’ most recent treatment location rather than the location of the episode that triggered the survey in the first place. In terms of message management, care team members replied to 24.6% (9057/36,838) of the messages and marked 40.9% (15,069/36,838) of the messages as completed (marked as read) without replying, and 34.5% (12,712/36,838) of the messages were further documented in a clinical note within the EHR by following the process shown in Figure 2. Regarding volume, 61.6% (22,692/36,838) of the alerts were yellow, 13.3% (4896/36,838) were red-yellow, 12.4% (4561/36,838) were red, and 12.7% (4689/36,838) were not labeled with a color, which are referred to here as *unlabeled alert*.

When comparing the status of the alert against the alert level, we saw an increase in the care team documentation activity as the alert level increased, which provides a care team–based validation signal of the effectiveness of the alert threshold. The lowest level of alerts being documented were the unlabeled alerts with 27.68% (1298/4689) of all unlabeled alerts documented, whereas the highest level of alerts being documented were the red alerts with 56.83% (2592/4516) of them being documented in the EHR.

We have analyzed the turnaround time to respond to alerts by three different time windows in Table 2: (1) during business hours (8 AM to 6 PM on Monday to Friday), (2) outside business hours (during weekdays between 6 PM and 8 AM the next morning, excluding Sunday to Monday and Friday to Saturday), and (3) over the weekend (after 6 PM on Friday until 8 AM on Monday). The median response time during business hours was 1 hour, with response time varying by alert severity; red alerts had a response time of under an hour, and unlabeled alerts had a median response time of 2 hours. Alerts received outside business hours took longer to review and had a median response time of 6 hours, showing a decrease in response time with increasing severity levels, indicating that care team members used the color label as an effective triaging mechanism, responding to the most critical alerts faster.

**Table 2:** Summary of alert type by message status and alert arrival window.

			Unlabeled alert (N=4689)		Yellow alert (N=22,692)		Red-yellow alert (N=4896)		Red alert (N=4561)		Total (N=36,838)
**Message status, n (%)**											
	Completed	3026 (64.5)		9718 (42.8)		1630 (33.3)		695 (15.2)		15,069 (40.9)	
	Replied	365 (7.8)		6040 (26.6)		1378 (28.1)		1274 (27.9)		9057 (24.6)	
	Documented	1298 (27.7)		6934 (30.6)		1888 (38.6)		2592 (56.8)		12,712 (34.5)	
**Time window, n (%)**											
	During business hours	2685 (57.3)		12,159 (53.6)		2597 (53)		2534 (55.6)		19,975 (54.22)	
	Outside business hours	757 (16.1)		3795 (16.7)		836 (17)		745 (16.3)		6160 (16.72)	
	Over the weekend	1247 (26.6)		6738 (29.7)		1436 (29.3)		1282 (28.1)		10,703 (29.1)	
**Time window, median response time in hours**											
	During business hours	2		1		1		0.5		1	
	Outside business hours	9		6		7		2		6	
	Over the weekend	40		23		2		21		22	

## Discussion

### Principal Findings

This study is one of the first studies to report findings on the design, implementation, and operationalization of a PRO critical results management and communication system in cancer care. Although many studies have reported the development of PRO systems, none have focused in depth on the management of results. Our findings suggest that there ought to be a mechanism in place to handle critical patient-reported results in a timely manner so that patients can discuss their symptoms with the care team. To this end, we enhanced an existing secure messaging system to facilitate asynchronous communication between patients and their care teams. Given that the care team can vary in size and composition owing to continually changing shifts, our findings show a median 5-person care team; similar findings have been reported by others [48,49]. It was important to develop a solution where the entire care team had visibility into the prior interactions with the patient to seamlessly pick up the conversation where another care team member left off. The flagging feature was useful for notifying senior team members of a message needing their attention. Although used less frequently, the ability to reassign patients to different care teams allowed for a smooth hand-off between teams.

### Setting Clinically Meaningful Alert Thresholds

It was critical to establish clinically meaningful thresholds on a patient cohort, setting a baseline definition of what “normal” symptoms might look like on any given day after a treatment episode. The alert thresholds were a highly debated topic and were revisited many times throughout the post–go-live period of each instrument. The decisions were made within each program work group, where the teams discussed the implications of turning on the alerts and anticipated impacts on workload. The decisions were based on the experience of handling reports of symptoms after treatment episodes targeted for alerting. Clinical care teams consisted of nurses who were well versed in collecting symptom data from patients via phone calls; therefore, they knew which symptoms they would hear on a specific day after an event such as surgery and made their decisions based on clinical judgment. As health care systems learn about patient outcomes over time, it is important to be able to adjust the thresholds. Alert rates were reviewed by staff through summary dashboards, allowing team members to reflect on alert workload burden of staff and determine mitigation strategies. By reviewing the dashboards, management noticed a high alert rate for symptoms such as pain reported the day after a surgical event, which was determined to be a normal clinical event. As a result, alert rules were adjusted to not fire for specific questions within 2 to 3 days after surgery. Setting thresholds such as “red” and “yellow,” which indicate severe and moderate symptoms, respectively, created visual indicators for the care team members within the subject as well as the body of the message. Notably, we saw a substantial difference in response times for the alerts that were marked as “red,” suggesting that the alert color label was effectively used as a triage mechanism. With this approach, by focusing on the alerts that are marked as “red,” we can reduce clinician burnout by minimizing the cognitive load associated with reading patient messages.

### Importance of PRO Governance

Similar to other studies on the importance of PRO governance [50], this study also shows that the governance committee (eForms Committee, 35 individuals) was instrumental in the design and implementation of PROs. This committee met monthly to discuss best practices of the overall system design and implications of new feature releases and made decisions establishing clinically meaningful alert thresholds across different patient cohorts. Clinicians, nurses, patient education specialists, and administrative staff served as collaborative thought leaders consistently striving to minimize patient burden and staff alert fatigue through critical assessment of the alert thresholds that were set. The clinically focused PRO work group (40 individuals) met on a more regular biweekly basis to define staff workflows and responsibilities, provide feedback on system design, align alerting with the existing messaging workflows, develop educational material for patients, create training material for staff, and reflect on the summary data presented via real-time dashboards. Policies were established around the roles and responsibilities of the care team members to ensure that messages were responded to in a timely manner. Decidedly, nursing and office staff were instrumental in reviewing responses, triaging, and responding directly to patients, resonating with similar findings of nurse-led patient engagement programs [17,51-54]. With the exception of 1 program, which had dedicated staff reviewing patient messages on weekends, patients were informed that the mailbox was unattended outside of regular business hours and that they were encouraged to call their physician in case of urgent symptoms.

### PRO Integration Into Clinical Workflows

Integrating PROs into clinical workflows has always been a challenge, and having clinicians review and act on the data is yet another challenge. Establishing an alerting system, notifying clinicians of only clinically meaningful patient responses via alerts is a step toward a better direction, where clinicians only need to review alerts if they are deemed clinically significant by clinical expert consensus. Given the emerging problem of clinician burnout, partially caused by information overload, by using this alerting system, the care teams reviewed 7.82% (36,838/470,841) of the patient responses and eliminated the need to review over 92.28% (434,003/470,841) of the responses, while maintaining the collection of valuable PRO data to study long-term patient outcomes in response to treatments. Having a seamless mechanism in place to communicate with patients within the same workflow is yet another step in the right direction and is aligned with findings from the literature [27]. Not only does it signal to the patient that they are being constantly cared for, encouraging them to keep completing their assessments, but it can also be used to address the symptoms early, preventing any unnecessary emergency room visits.

### Future Research Opportunities

Although PRO data can be a valuable tool for shared decision-making and bridging the care gap for in-between visits, the data are only available if patients complete their assessments. The adoption of remote monitoring programs during the COVID-19 pandemic [55-57] exposed the digital divide created by programs solely relying on digital interventions. Patients who are not as comfortable with technology or those whose primary language is different from the language of the survey instrument may be less likely to complete their symptom assessments. As we scale PRO-based remote symptom monitoring programs, we must consider studying the sociotechnical aspects of a wholly digital intervention. There are several implications for future research opportunities with respect to setting meaningful alert thresholds appropriately. At MSK, the deliberations around setting clinically meaningful thresholds evolved over time and, ultimately, were decided through agreement between nursing staff accountable for responding to alerts and physicians responsible for patient outcomes. In addition, it would be interesting to analyze the impact of patient characteristics, such as demographics, disease stage, or disease type, on alert response patterns and communication with care team members. As we accumulate more robust PRO data sets and monitor clinician triage, there is an opportunity to build machine learning models to predict when patients will need interventions based on their responses to specific PROs, response patterns, and clinical context such as disease stage and progression. In addition, automated artificial intelligence–based chatbots can be developed to facilitate conversations with patients, reducing the burden on nursing staff. Further studying follow-up activities of nurses in EHRs such as referrals, medication orders, or communication with other care team members can inform the refinement in the chatbot responses to patients.

### Limitations

This study has some limitations. First, we report findings from one institution, which may not be generalizable across all settings. In addition, because we have an in-house–developed patient engagement system, we had the flexibility to design and implement an alert management system that was best suited to the care team workflows, which may not be a flexibility affordable to other health care institutions. Moreover, the care teams that opted to implement alerting features for their PRO programs were highly engaged in the system development life cycle and provided ample feedback throughout the process, which may not apply to institutions with limited resources. System design features for most of the functionalities were informed through discussions with a pilot work group and were qualitative in nature, and we did not perform a formal quantitative assessment.

### Conclusions

By developing a critical symptom alerting and communication system, we designed a system supporting the real-time delivery of critical results based on PRO data to appropriate care team members, including the ability for a patient and clinical staff to communicate in a nonstructured, text-based, secure communication format about the alert. We were able to standardize the processing of patient-generated alert messages, enabling the presentation of clinically meaningful PRO data within clinical workflows in a standard format, and monitor response times by clinical staff. This allowed us to set an appropriate patient expectation for a response time frame by their care team members or provided alternate communication guidance specific to each patient and the surgical procedure they underwent.

## Appendix

Multimedia Appendix 1 Summary of questionnaires using the alerting features implemented in this study.

## Abbreviations

EHR: electronic health record
FITT: Fit between Individuals, Task, and Technology
MSK: Memorial Sloan Kettering
PRO: patient-reported outcome
