# Supplementary material for: Managing Critical Patient-Reported Outcome Measures in Oncology Settings: System Development and Retrospective Study
Source: JMIR Med Inform. 2022 Nov 3;10(11):e38483. doi: 10.2196/38483 (PMC9672998; doi:10.2196/38483)
Supplement: Multimedia Appendix 1 [file medinform_v10i11e38483_app1.docx]

**Multimedia Appendix 1**

**Supplementary Data**

**Table S1: Summary of Questionnaires that utilized alerting features implemented in this study.**

| Survey Name | Description and Use | Scope |
| --- | --- | --- |
| Acute Side Effects From Breast Radiation | Symptom tracker for patients with Breast radiation treatment, standard of care. This survey was administered to all patients undergoing breast radiation treatment, median patient age was 58, average survey completion rate was 48%. | Breast Radiation Oncology |
| Connected Care Questionnaire | Ten-day symptom questionnaire post hospital inpatient discharge episode, standard of care. This survey was administered to all patients recruited to the Connected Care program via standard hospital discharge protocols for inpatient stays for the medical oncology services listed under scope. Median patient age was 64, average survey completion rate was 32% | Thoracic and Gastrointestinal Medical Oncology |
| COVID-19 Symptom Questionnaire | Fourteen-day survey given to COVID-19 positive all patients post inpatient discharge or positive COVID-19 result in the outpatient setting, surveys were administered as part of standard of care procedures for all eligible patients. Median patient age was 60 and average survey completion rate was 45%. | All services |
| Daily Symptom Assessment | Ongoing symptom tracker for the Insight Care Program, monitoring chemotherapy patients enrolled in the program. Median patient age was 67 and average survey completion rate was 58%. | Breast, Head & Neck, Gynecologic Medicine and, Plastics |
| Employee COVID-19 Recovery Tracker | Fourteen-day survey given to all COVID-19 positive employees post positive result in the outpatient setting, standard of care. Median patient age was 37 and average survey completion rate was 34%. | Employee Health Service |
| Novel Coronavirus (COVID-19) Screening Questionnaire | COVID-19 symptom screening questionnaire given to all patients 1 day before their planned outpatient in-person appointment. Median patient age was 63 and average survey completion rate was 34%. | All outpatient services |
| Recovery Tracker (5 Day) | Five day post ambulatory care surgery event, monitoring a smaller subset of symptoms for less complex procedures for all qualifying ambulatory surgery cases at. Median patient age was 61 and average survey completion rate was 41%. | Ambulatory Surgery |
| Recovery Tracker (10 Day) | Ten day post ambulatory care surgery event, monitoring a larger subset of symptoms for more complex surgical procedures done at an ambulatory care setting. Median patient age was 56 and average survey completion rate was 35%. | Ambulatory Surgery |
